# A case of stromal sarcoma of the prostate with the incidental detection of prostate adenocarcinoma

**DOI:** 10.1016/j.eucr.2025.102963

**Published:** 2025-01-28

**Authors:** Shang Xu, Xinning Wang, Wei Jiao

**Affiliations:** Department of Urology, The Affiliated Hospital of Qingdao University, Qingdao, Shandong Province, China

**Keywords:** Case report, Prostate adenocarcinoma, Prostate stromal sarcoma

## Abstract

Prostate stromal sarcoma is very rare among patients with prostate cancer, accounting for <0.1 % of prostate malignancy. Here, we report a case of prostate stromal sarcoma combined with incidental adenocarcinoma with normal serum PSA after radical prostatectomy. This case is unique in that the occurrence of incidental adenocarcinoma might interfere with the postoperative follow-up and treatment. We can only draw from the experiences of case reports and retrospective analysis. This article describes a presentation of a rare tumor case and a review of the literature.

## 1 Introduction

Prostate sarcoma is an uncommon kind of prostate cancer, with the incidence of primary prostate sarcoma among all prostate cancer diagnoses being less than 0.1 %.^1^ Prostate sarcoma can manifest as different pathological types since it originates from nonepithelial mesenchymal tissues such as smooth muscle, blood vessels, and nerves. Prostate stromal tumors, which originate from the mesenchymal components of the prostate, were first classified by Gaudin in 1998.^2^ Prostate stromal sarcoma, which originates from mesenchymal components of the prostate, is extremely rare among prostate sarcomas,^3^ accounting for only 5.2 % in patients over 25 years of age.^4^ Fewer than 100 cases of primary prostate stromal sarcoma have been documented in the literature to date. Here, we report a case of primary prostate stromal sarcoma with primary prostate adenocarcinoma in a 50-year-old man presenting with normal PSA and a large prostate mass.

## 2 Case report

**Fig. 1:**
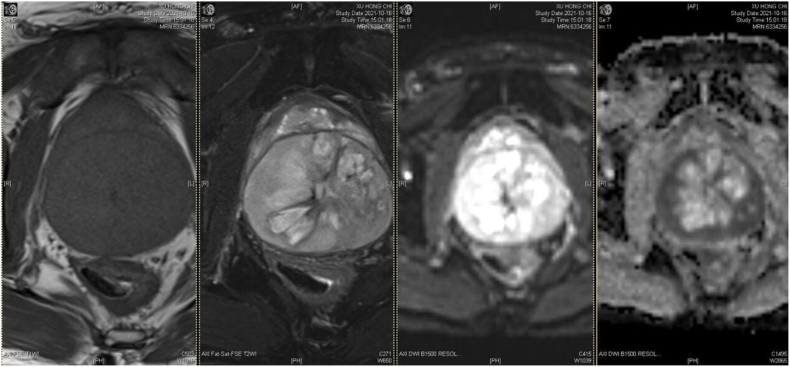
mpMRI multiparametric MRI, DWI diffusion weighted MRI, ADC Apparent diffusion coefficient.

**Fig. 2:**
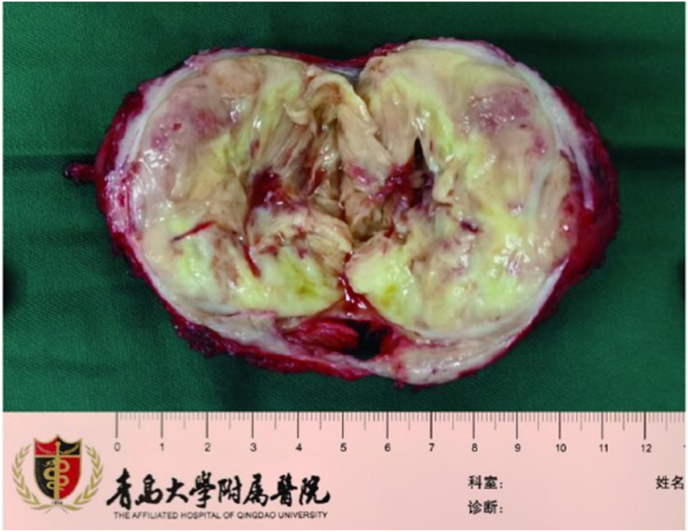
Gross examination showed a 9-cm mass with necrotic areas.

A 50-year-old asymptomatic man presented to the Affiliated Hospital of Qingdao University in October 2021 with a prostate mass detected at a health checkup. The preoperative serum tPSA was 2.32 ng/ml; f/t was 0.10. A rectal examination revealed a large, benign-feeling prostate mass. The MRI signal showed a long T1, a mixed T2, and hyperintensity on DWI (Fig. 1). PET/CT did not detect any suspicious metastasis. After clinical confirmation of the sarcoma by prostate needle biopsy, the patient underwent laparoscopic radical prostatectomy. The size of the surgical specimen was 9.0∗8.0∗8.0 cm, and the size of the center mass was 9.0∗6.0∗6.0 cm with a large necrotic area (Fig. 2). However, the patient did not consent to neoadjuvant or adjunctive treatment due to a fear of chemotherapy. PET/CT has advantages in differentiating the presence of distant metastases, but it is difficult to detect local recurrence due to the excretion of radiotracer in the urine. One year after the operation, we first conducted a chest and whole - abdomen CT examination, and the results showed no signs of recurrence. However, during the review at the 19th month after the operation, a pelvic mass with a diameter of about 9 cm was detected. Subsequently, the patient underwent a pelvic mass resection, and the post - operative pathology revealed metastatic prostate stromal sarcoma. Despite our recommendation for chemotherapy, the patient still refused to receive it after the operation. Later, widespread intra - abdominal tumor metastasis occurred at the 28th month after the operation, and the patient unfortunately passed away at the 34th month after the operation.

**Fig. 3:**
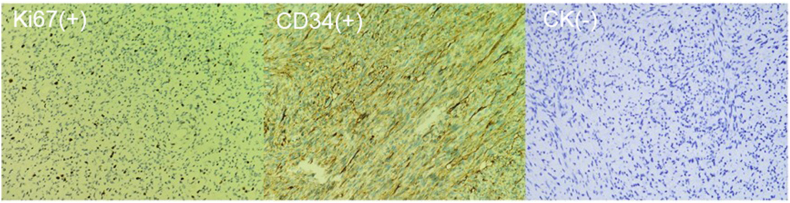
Immunohistochemical staining of Ki67, CD34 and CK in prostate stromal sarcoma.

Postoperative pathology revealed that the tumor was a prostate stromal sarcoma. The tumor tissue configuration was monophasic and composed of sarcomatoid spindle cells, which were arranged in interlacing fascicle patterns, similar to fibrosarcoma. Tumor cells showed moderate to severe atypia, and large necrotic areas were present among the tumor tissue. Pathological nuclear division was also observed in the hotspot (>15/10HPF). Giant cell formation and heterologous differentiation were not detected in the tumor tissue. Immunohistochemistry of the prostate stromal sarcoma showed that the expressions of ki67 and cd34 were positive, while those of CK were negative (Fig. 3). Prostate stromal sarcoma genetic testing showed CDK4, MDM2, GLI1, IFITM3, ATRX, FRS2, THAP2, and TMEM4 mutations, low TMB and MSI, and lower expression of PD-L1.

Moreover, a piece of prostate adenocarcinoma with a size of 0.3∗0.2 cm was detected in the normal prostate tissues around the stromal sarcoma by accident in wax from the specimen's surgical margin. The Gleason score of the prostate adenocarcinoma was 3 + 4 = 7. Immunohistochemistry of the adenocarcinoma showed that the expression of P504S was positive, while those of P63 and HCK were negative.

**Fig. 4:**
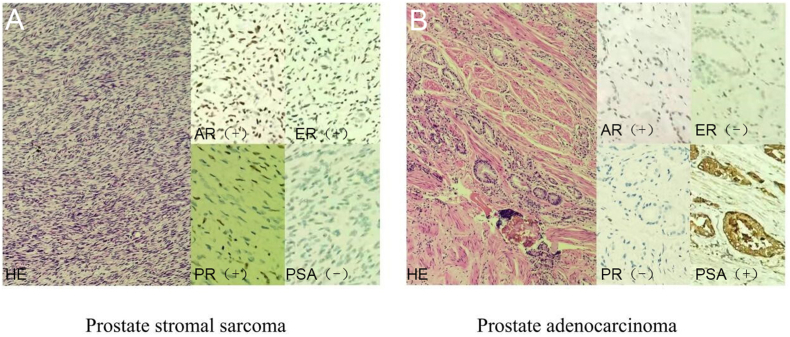
Representative example of prostate histology and immunohistochemical staining of AR, ER, PR and PSA in prostate stromal sarcoma and prostate adenocarcinoma.

We also detected the expressions of PSA, androgen receptor (AR), estrogen receptor (ER), and progesterone receptor (PR) in the tumor. The expressions of AR, ER, and PR was positive in the prostate stromal sarcoma, while that of PSA was negative. In addition, the expressions of PSA and AR were positive in the prostate adenocarcinoma, while those of ER and PR were negative (Fig. 4).

## 3 Discussion

There are many pathological types of prostate sarcoma. Because of its rarity and histological diversity, it is still not a reliable diagnostic modality. The most common initial symptoms and chief complaints are urinary obstructions and perineal pain among Chinese and American patients.^5^

Pathologically, differential diagnosis between prostate stromal sarcoma and sarcomatoid carcinoma is needed. Sarcomatoid carcinoma originating from the prostate is accompanied by high‐grade adenocarcinoma cells among tumor tissues and the expression of CK and PSA. Sarcomatoid carcinoma is a special type, but it is still a kind of adenocarcinoma with poor differentiation. In most sarcomatoid carcinoma cases, we can detect some high-grade adenocarcinoma component among the tumor tissues. However, we could not detect any high-grade adenocarcinoma cells or CK/PSA-positive cells in tumor tissues in the present case. We only found a low-grade adenocarcinoma in the normal prostate tissues. Additionally, the histochemical findings did not support a diagnosis of sarcomatoid carcinoma. Hence, this case was diagnosed with prostate sarcoma.

The subtypes of prostate sarcoma include leiomyosarcoma, rhabdomyosarcoma, malignant fibrous histiocytoma, and unclassified sarcoma.^3^ Rhabdomyosarcoma is most commonly found in children. Leiomyosarcoma is most commonly found in adults and accounts for approximately 30 % of prostate sarcomas.^4^ According to one study, leiomyosarcoma subtypes lead to worse survival than non-leiomyosarcoma subtypes.^6^ Unclassified sarcoma can be further divided into primary stromal sarcoma and stromal tumors of uncertain malignancy potential based on the invasive area of mesenchyme and mitosis and the extent of mesenchyme overgrowth.^7^^,^^8^ This is the first report of primary prostate stromal sarcoma combined with prostate adenocarcinoma. Histologically, prostatic stromal sarcomas are much easier to diagnose as neoplastic because their overtly malignant features are more easily distinguished from florid stromal hyperplasia. Stromal sarcomas often show greater cellularity, greater cytological atypia, easily identifiable mitotic activity, and tumor cell necrosis.^9^

The ideal treatment paradigm for prostate sarcoma is still unknown due to its rarity, precluding large randomized studies. However, small cohort studies could be a valuable tool to study this disease. As reported, curative surgery with negative margins is one of the most significant prognostic variables.^5^ Ding and colleagues reported 41 Chinese cases whose median survival was 18.6 months.^5^ This result is lower than those of other studies referenced in this article due to a more active radical therapeutic surgery, including cystoprostatectomy and pelvic exenteration, being more frequently adopted in the USA. In the case we reported, the patient only underwent surgical resection without systemic chemotherapy and survived for 34 months.

Although extirpative surgery is the first option, especially in organ-confined disease, multimodal therapy combined with surgery and adjuvant treatment could exhibit good clinical efficacy.^10^, ^11^, ^12^, ^13^ Multidisciplinary teams could play an essential role in diagnosing patients, planning treatment, and improving their quality of life.^14^ Musser and colleagues updated the Memorial Sloan Kettering experience of treating prostate sarcoma and endorsed neoadjuvant therapy in select cases to ensure complete resection.^11^ More recently, Ball et al. also published their data that showed that neoadjuvant radiotherapy and radiosensitizing chemotherapy before surgery could significantly improve the recurrence-free survival and CSS of patients with prostate sarcoma.^10^ All eight patients underwent surgical resection and neoadjuvant radiation, where six had concurrent chemotherapy and four received intraoperative radiation. The median OS and CSS were 67.8 months, with actuarial OS and CSS rates of 100 % at 1 year, 75 % at 2 years, 62.5 % at 3 years, and 62.5 % at 5 years. However, only one patient achieved complete resolution. This result also demonstrates the poor prognosis of sarcoma. Most sarcoma patients have an extremely short survival time, even among those who undergo surgical resection of the tumor.^15^ Some clinical trials using novel target agents, such as regorafenib and nivolumab, to treat soft tissue sarcoma have been established.^16^^,^^17^ This research might help to provide a new neoadjuvant or adjunctive therapy scheme for adult prostate sarcoma in the future. Furthermore, immunochemically, prostate stromal sarcoma normally expresses PR.^11^ Some cases might not express ER. However, AR, ER, and PR were all positive in this case. This suggests that patients diagnosed with prostate stromal sarcoma might benefit from ADT or endocrine therapy. As previously reported, the overexpression of ER and PR might be associated with unfavorable prostate adenocarcinoma outcomes.^18^^,^^19^ This also indicates that endocrine receptors might play the same role in prostate sarcoma and advanced cancer. The mechanism needs to be studied in the future.

Optimization of the systemic treatment is also needed to control metastasis, which is the predominant pattern of relapse.^13^ Distant metastasis is significantly associated with shorter survival.^5^^,^^13^ Early metastases are common in patients with prostate sarcoma, and typical sites include the lung, liver, and bone.^4^^,^^20^ However, our case indicated that multidisciplinary treatment should be used to thoroughly locate the source of metastasis in cases with both sarcoma and adenocarcinoma. If metastasis in bone, lymph nodes, or other organs is detected, a careful histological examination of the metastasis should be performed to clarify whether it is due to prostate sarcoma or adenocarcinoma, as the misjudgment of the origin of the metastasis could result in incorrect treatment and affect patient survival.

## 4 Conclusion

Prostate sarcoma is a rare kind of prostate tumor. The differential diagnosis between prostate stromal sarcoma and sarcomatoid carcinoma is indispensable. Additionally, this case also demonstrated that incidental prostate adenocarcinoma is possible. Hence, careful pathological examination is necessary to exclude incidental prostate adenocarcinoma, especially in those with abnormal serum PSA. Furthermore, a rigid follow-up plan should be made to confirm which tumor relapses or needs further treatment.

## Ethics approval and consent to participate

This article was approved by the Ethics Committee of the affiliated hospital of Qingdao University.

## Consent for publication

All author consent for this article publication.

## Availability of data and material

N/A.

## CRediT authorship contribution statement

**Shang Xu:** Writing – original draft. **Xinning Wang:** Data curation. **Wei Jiao:** Writing – review & editing, Supervision.

## Funding

N/A.

## Competing interests

The author declare that there no competing interests.
